# Luminal A Breast Cancer: How Feasible is Omitting Axillary Dissection Without Neoadjuvant Therapy

**DOI:** 10.1155/2022/8284814

**Published:** 2022-07-30

**Authors:** Cemil Yüksel, Bülent Aksel, Lütfi Doğan

**Affiliations:** University of Health Science, Ankara Abdurrahman Yurtaslan Oncology Training and Research Hospital, Ankara, Turkey

## Abstract

**Background:**

Luminal A breast cancer has a good prognosis and the criteria for adjuvant and neoadjuvant chemotherapy (NAC) are not clear. The aim of this study was to present our results of upfront surgery and long-term survival in luminal A tumors as well as the rates of protection from axillary dissection. *Material and Methods*. 271 Luminal A breast cancer patients who had operated at our center were evaluated retrospectively. In patients with 2 or less sentinel lymph node (SLN) positivity who did not receive neoadjuvant therapy and underwent breast-conserving surgery, axillary lymph node dissection was omitted (OAD). Axillary lymph node dissection (ALND) was performed in patients with positive SLN who did not meet these criteria (axillary dissection after sentinel/ADAS).

**Results:**

While Sentinel Lymph Node Biopsy (SLNB) was performed in 212 (77.9%) patients, SLNB + Axillary Dissection (AD) was performed in 58 (21.3%), and direct axillary dissection was performed in 1 (0.8%) patient. OAD was applied to 18 (23.6%) of the positive patients. *Discussion/Conclusions*. ALND rates are still strikingly high in luminal A breast cancer treatment, despite the disease's milder clinical course. In order to avoid complications of axillary dissection, patients should be considered for NAC as much as possible. Novel neoadjuvant or other therapy options are also required.

## 1. Introduction

Luminal A breast cancer is the most common subtype of breast cancer; that is, estrogen (ER) and/or progesterone (PR) positive, human epidermal growth factor receptor 2 (HER2) negative, has low proliferative activity and a good prognosis [1]. However, the selection criteria for adjuvant and neoadjuvant chemotherapy (NAC) are unclear for this subtype. Various studies have shown that lymph node positivity is not an indication for chemotherapy. [2–4] This complicates the management of luminal A breast cancer. In general, tumors with an aggressive course respond rapidly and dramatically to chemotherapeutic agents while nonaggressive ones have a slower response [5]. In that respect, NAC has become almost standard in triple-negative (TN) and HER2 positive cancers regardless of stage [6].

On the other hand, ambiguities continue regarding the neoadjuvant management of luminal A tumors with a milder clinical course. Even at a locally advanced stage, luminal A tumors may not respond well to NAC and considering that risk, the physician may opt for upfront surgical treatment. Besides, the feasibility of ACOSOG Z0011 criteria in patients with operable luminal A breast cancer also suggests starting the treatment with surgery. According to the criteria, completion axillary dissection is unnecessary for patients who do not receive NAC but undergo breast-conserving surgery (BCS) and have 2 or less positive sentinel lymph nodes (SLNs). [7] However, if there is residual disease in the SLN after NAC, axillary lymph node dissection (ALND) is required. Performing an upfront surgery and applying the ACOSOG Z0011 criteria ensures avoiding ALND in patients with lymph node metastasis who are considered poor candidates for NAC [8]. This study presents our axillary intervention and long-term survival results of upfront surgery in luminal A breast cancer.

## 2. Materials and Methods

This retrospective cross-sectional study involved luminal A breast cancer patients who were operated on in our center between January 2017 and March 2021. Luminal A subtype was defined as having ER and/or PR positive, HER2 negative, and Ki67 index <14%. Patient data including surgery and pathology reports, hormone receptor status, demographic characteristics, tumor size, location, grade, stage, surgical intervention type, neoadjuvant and adjuvant therapies, overall survival (OAS), disease-free survival (DFS), and locoregional recurrence-free survival (LRFS) were recorded retrospectively. We evaluated sentinel lymph node biopsy (SLNB) and axillary surgical approaches to determine their rates of omission of axillary dissection (OAD). Women with newly diagnosed nonmetastatic Luminal A breast cancer who were given adjuvant hormonal therapy combined with mastectomy or breast-conserving surgery + radiotherapy were included in the study. Our investigation excluded patients with distant metastasis, male breast cancer, malignancy other than breast cancer, neoadjuvant or adjuvant therapy for another malignancy, carcinoma in situ (CIS), previous breast surgery, and unavailable data. The study population consisted of 271 patients.

**Figure 1:**
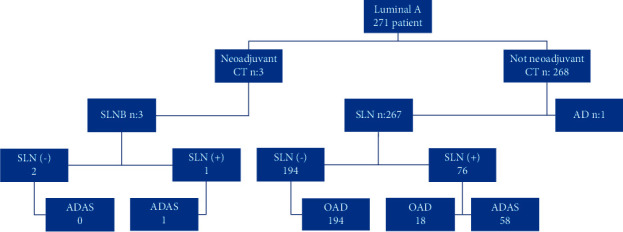
Patients' flow chart.

Patients who underwent breast-conserving surgery (BCS) and mastectomy based on indications received radiotherapy. SLNB was performed in those without clinical findings of axillary lymph node metastasis. The ACOSOG Z0011 guideline was applied in cases meeting the relevant criteria. We opted for OAD in patients who did not receive neoadjuvant therapy, underwent BCS, had 2 or less SLN positivity, and had direct SLN negativity. SLNB + ALND, i.e., axillary dissection after SLNB (ADAS), was preferred in SLN-positive patients who did not meet these criteria (Figure 1).

The pathology clinic performed the histopathological and immunohistochemical examinations. Hormone receptor status testing was performed and recorded as positive (expression rate ≥1%) or negative. HER2 positivity ≤1 was considered negative, 2 HER2 was eligible for fluorescence in situ hybridization (FISH), and 3 HER2 was considered positive.

We also obtained the patients' follow-up data. Disease-free survival (DFS) was considered the time from the date of surgery to the date of local recurrence or distant metastasis; locoregional recurrence-free survival (LRFS), from surgery to locoregional recurrence; and overall survival (OAS), from the diagnosis of breast cancer to death.

### 2.1. Statistical Analysis

We used the SPSS 11.5 software package for statistical analysis. Quantitative variables were expressed as the mean ± standard deviation and median (minimum-maximum), and qualitative variables as number (percentage). The Shapiro–Wilk test was performed for normality. We conducted the Mann-Whitney *U* test to compare differences between the categories of a qualitative variable in terms of a quantitative variable when the data was not normally distributed. The chi-square and Fisher's exact tests were used to examine the relationship between two qualitative variables. The DFS, LRFS, and OAS were estimated by the Kaplan–Meier method and compared with the log-rank test. A *p* ≤ 0.05 was considered statistically significant.

## 3. Results

The study population comprised 271 patients meeting the inclusion criteria. Throughout our investigation, only 3 patients with a new diagnosis were shifted to NAC. The median age of all patients was 57 (28–83), and the mean tumor diameter was 20.59 mm. 207 (76.3%) had invasive ductal carcinoma, 37 (16.3%) invasive lobular, 19 (7%) mucinous, 7 (2.5%) tubular, and 1 (0.6%) mixed type. 45.2% of the tumors were located in the upper outer quadrant. Tables 1 and 2 show the general characteristics of the patients.

We performed mastectomy in 100 (36.9%) patients, oncoplastic BCS in 137 (50.5%), and conventional BCS in 34 (12.6%). 212 (77.9%) patients underwent SLNB, 58 (21.3%) ADAS, and 1 (0.8%) patient upfront ALND. 194 (71.3%) of the patients who underwent SLNB were negative; 76 (28.7%) were positive. We preferred OAD in 18 (23.6%) of the positive patients and performed ADAS on the rest [58]. 16 of the OAD patients had 1 metastatic lymph node, and 2 had 2 nodes. Axillary metastases were detected in only 13 out of 58 patients who underwent axillary dissection.

3 patients received preoperative NAC. 2 of these had negative SLNB; 1 was SLN positive and proceeded to ALND. One of these 3 patients underwent mastectomy due to the multifocal tumor and the other 2 underwent oncoplastic BCS. Local recurrence, distant metastasis, and mortality did not occur in these patients. We made no relevant comparisons here since only 3 patients had received NAC. Of the 58 ALND patients, 29 had undergone mastectomy and were SLN positive, and 29 had undergone BCS and had >3 SLN positivity. We performed upfront ALND without SLNB in 1 patient. Our investigation of the relationship between the demographic, histopathological, and clinicopathological characteristics of the patients with positive SLNB and OAD revealed that OAD was significantly more common at the T1 stage (*p* < 0.001). However, there was no statistically significant relationship between these two groups regarding other variables (Table 3).

The patients' mean follow-up period was 33.08 months. Concerning OAS, 3 patients (1.1%) died. The mean DFS was 32.74 months and the LRFS was 33 months. Local recurrence developed in 1 (0.4%) patient and distant metastasis in 4 (1.5%) patients. Clinical *T* stage, choice of axillary dissection, lymphovascular invasion (LVI) and distant metastasis stood out as the factors affecting OAS with statistical significance.  Table 4 summarizes the survival analysis of our study population.

## 4. Discussion

The management of breast cancer has increasingly become targeted and individualized. Gene expression profiling allows the determination of breast cancer subtypes [9], which has become paramount in treatment planning. [10] Although hormone receptor-positive patients with low HER2 and Ki67 expression have longer survival times, further research is required on the axillary surgical approach in these patients as the relevant published literature is insufficient. Most articles concerning luminal A breast cancer are focused on adjuvant chemotherapy. [11–13] On the other hand, there is an ever-growing tendency toward OAD due to ALND complications that impair quality of life. Novel NAC modalities may ensure more frequent OAD.

Luminal A breast cancers generally have better long-term outcomes than other subtypes [14]. However, clinicians generally tend to avoid NAC in treating this subtype due to its low efficacy. [15] Especially in patients with SLNB positivity detected beforehand, NAC allows axillary effacement, SLNB preventing ALND and its complications such as movement limitation, lymphedema, and pain. [16] However, studies cite the rate of axillary effacement after NAC in luminal A breast cancers at <25%. [17] Besides, high resistance to NAC in luminal A breast cancer necessitates its avoidance in some cases, rendering ALND inevitable. Studies on neoadjuvant endocrine therapy in early-stage luminal A breast cancer mainly concern the rates of BCS and mastectomy without any evaluation of the extent of surgery and axillary intervention. [18] In the present study, only 3 patients had received NAC; **76** of the patients without NAC were SLN-positive, and 58 underwent ALND. We think by expanding the indications to NAC, OAD rates can increase, preventing ALND complications.

In our study, the SLNB negativity rate among our patients was 72.6%. Axillary dissection was omitted in these patients, but a higher OAD rate should be targeted in this breast cancer subtype with a milder clinical course. OAD was feasible only in 18 of our 76 SLN-positive patients (23.6%) as per the ACOSOG Z0011 criteria, whereas 58 patients not meeting the criteria underwent ALND. As a result, the total rate of patients who had to undergo ALND was 21.7%. Half of these consisted of SLN-positive mastectomized patients, and the other half consisted of patients with BCS having 3 or more SLN positivity. Tullberg et al. reported a 17.6% metastasis rate for 4 or more SLN positivity in their series of node-negative luminal A breast cancer patients. [19] In contrast, Herr et al. indicated a 34.1% metastasis rate for 3 or more SLN positivity in SLN-positive patients. [20] Approximately 20% of luminal A breast cancers are diagnosed at stages N_2_ and N_3_. [21] These data suggest that the overall rates of OAD need improvement. As further studies investigating OAD in sentinel lymph node macrometastases in patients undergoing mastectomy and receiving NAC materialize [22], the ongoing reluctance to administer NAC for luminal A tumors will potentially wane. Nevertheless, the current OAD rates for SLN-positive luminal A breast cancer patients without NAC are unsatisfactory. Until conclusive literature remarkably influences the clinical approach, the patients should be thoroughly evaluated for NAC candidacy.

Our comparison of OAD and ADAS groups of SLNB-positive patients yielded statistically significant results only for the T1 stage, where the OAD rate was higher. We observed no significant difference between the two groups regarding local recurrence, distant metastasis, and survival times. Still, OAD proved beneficial via preventing ALND complications, suggesting that surgical morbidity was crucial in OAD and ADAS evaluation. Therefore, more effective screening programs may improve early-stage cancer detection and, in turn, OAD rates. Another alternative is to apply the ACOSOG Z0011 criteria after NAC. Besides, future research may result in new NAC agents to promote axillary effacement. We believe that this is a lengthy and painstaking process full of prospects.

Survival studies on invasive breast cancer have associated LVI with poor survival. [23, 24] In the present study, 17 (6.3%) patients had LVI in all patients also LVI patient's associated with poor prognosis in overall survival (*p*=0.025). However, there was no significant difference between OAD and ADAS in terms of LVI. Cheang et al. have reported a significant link between LVI and survival in hormone-positive patients without adjuvant therapy but not in those with adjuvant endocrine therapy [9]. In our study, only 1 (0.4%) patient developed a local recurrence and 4 (1.5%) patients developed distant metastases. In this breast cancer subtype with rare local recurrence and distant metastasis, ALND morbidity stands out as a decisive parameter in treatment since complications dramatically affect patients' quality of life. In our study population, advanced T-stage patients with distant metastases who underwent ALND had significantly poorer survival. Other studies have also noted these as poor prognostic factors. [25, 26] Cheang et al. had reported poorer survival for higher cancer grades, but our investigation did not yield a similar result, which may be explained by the fact that the former study had also included luminal B breast cancer patients. Besides, Cheang et al. also indicated poorer survival for larger tumor sizes, whereas we observed poorer survival in patients with advanced *T*-stage. Nevertheless, both studies have correlated axillary dissection with poor prognosis. Our study's single-center retrospective design has a limitation. However, we have managed to obtain significant results favoring OAD in our study population.

In conclusion, ALND rates are still strikingly high in luminal A breast cancer treatment, despite the disease's milder clinical course. In order to avoid complications of axillary dissection, patients should be considered for NAC as much as possible. Novel neoadjuvant or other therapy options are also required.

We suggest relevant future studies with larger populations for more accurate treatment planning favoring the omission of axillary dissection.

## Figures and Tables

**Table 1:** Patients' characteristics.

Variables		
Age	Mean ± SD	**56.74** **±** **10.89**

Adjuvant CT, *n*(%)	No	**139 (51.3)**
	Yes	**132 (48.7)**

Adjuvant endocrine therapy, *n*(%)	No	**0 (0)**
	Yes	**271 (100.0)**

Mortality, *n*(%)	No	**268 (98.9)**
	Yes	**3 (1.1)**

Local recurrence, *n*(%)	No	**270 (99.6)**
	Yes	**1 (0.4)**

Distant metastasis, *n*(%)	No	**267 (98.5)**
	Yes	**4 (1.5)**

SLNB : sentinel lymph node metastasis; SLN: sentinel lymph node; AD : axillary dissection; OAD : omitting axillary dissection; ADAS : axillary dissection after sentinel.

**Table 2:** Tumor characteristics.

Variables		
Tumor diameter	Mean ± SD	**20.68** **±** **12.48**

Tumor location, *n*(%)	Upper outer quadrant	**123 (45.2)**
	Lower outer quadrant	**42 (15.4)**
	Upper inner quadrant	**43 (15.8)**
	Lower inner quadrant	**29 (10.7)**
	Central	**9 (3.3)**

Pathology, *n*(%)	Invasive ductal carcinoma	**207 (76.3)**
	Invasive lobular carcinoma	**37 (13.6)**
	Mucinous	**19 (7)**
	Tubular	**7 (2.5)**
	Mixed	**1 (0.6)**

Grade, *n*(%)	**1**	**92 (33.8)**
	**2**	**153 (56.3)**
	**3**	**25 (9.2)**

cT, *n*(%)	**1**	**153 (56.3)**
	**2**	**110 (40.4)**
	**3**	**8 (2.9)**

SLN status, *n*(%)	OAD	**212 (77.9)**
	Positive-AD	**58 (21.3)**

Axillary intervention, *n*(%)	SLNB	**212 (77.9)**
	SLNB + Axillary dissection	**58 (21.3)**
	Axillary dissection	**1 (0.4)**

Positive lymph node counts in patients undergoing, *n*(%)	1 positive	**16 (88.8)**
	2 positive	**2 (1.2)**

SLN : sentinel lymph node; OAD : omitting axillary dissection; AD : axillary dissection; SLNB : sentinel lymph node biopsy.

**Table 3:** Comparison of patients with and without OAD.

Variables		OAD		ADAS		*p* value
		*N*	%	*N*	%	
Age	**<60**	**14**		**38**		**0.328**
	**≥60**	**4**		**20**		

Location	Upper outer quadrant	**6**		**32**		**0.336**
	Lower outer quadrant	**2**		**8**		
	Upper inner quadrant	**5**		**6**		
	Lower inner quadrant	**2**		**9**		
	Central	**1**		**1**		

cT	**1**	**14**		**13**		**<0.001**
	**2**	**4**		**39**		
	**3**	**0**		**6**		

cN	**0**	**17**		**55**		**0.793**
	**1**	**1**		**2**		
	**3**	**0**		**1**		

Multifocality	No	**16**		**55**		**0.375**
	Yes	**2**		**3**		

Grade	**1**	**5**		**9**		**0.379**
	**2**	**12**		**41**		
	**3**	**1**		**8**		

LVI	No	**18**		**49**		**0.075**
	Yes	**0**		**9**		

Local recurrence	No	**18**		**58**		**—**
	Yes	**0**		**0**		

Distant metastasis	No	**18**		**57**		**0.575**
	Yes	**0**		**1**		

OAD : omitting axillary dissection; LVI : lymphovascular invasion; ADAS : axillary dissection after sentinel.

**Table 4:** Survival analysis.

Variables		Survival			*p* value
		**2 **yr (%)	**4 **yr (%)	Time (mth)
				Mean ± SE
General		**99.2**	**98.0**	**53.53** **±** **0.26**	**—**

cT	**1**	**99.3**	**99.3**	**53.75** **±** **0.24**	**0.010 ** ^*∗*^
	**2**	**99.0**	**99.0**	**52.59** **±** **0.40**	
	**3**	**100**	**66.7**	**41.66** **±** **0.27**	

Age	**<60**	**99.4**	**99.4**	**52.73** **±** **0.27**	**0.313**
	**≥60**	**99.0**	**95.7**	**53.21** **±** **0.54**	

Grade	**1**	**98.8**	**95.7**		**0.537**
	**2**	**99.3**	**99.3**		
	**3**	**100**	**100**		

Axillary intervention	SLNB	**99.5**	**99.5**		**0.115**
	SLNB + Axillary dx	**98.2**	**90.0**		
	Axillary dx	**100**	**100**		

Axillary dx	Yes	**98.2**	**90.1**	**51.26** **±** **1.91**	**0.039 ** ^*∗*^
	No	**99.5**	**94.0**	**53.82** **±** **0.17**	

SLNB status	Negative	**99.4**	**94.5**		**0.114**
	Z0011 applied	**100**	**100**		
	Axillary dx	**98.2**	**90**		

SLNB-positive and Z0011 status	Z0011 applied	**100**	**100**		**0.450**
	Z0011 not applied	**98.2**	**90.0**		

pN	**0**	**99.4**	**99.4**		**0.086**
	**1**	**100**	**91.7**		
	**2**	**94.7**	**94.7**		

Adj. CT	No	**99.2**	**99.2**		**0.609**
	Yes	**99.2**	**97.0**		

Yr : year; Mth : month; SLNB : sentinel lymph node biopsy; Dx : dissection; Adj. CT : adjuvant chemotherapy.

## Data Availability

All data generated or analysed during this study are included in its supplementary material files. Further enquiries can be directed to the corresponding author.
